# Human papillomavirus (HPV) genotype distribution in penile carcinoma: Association with clinic pathological factors

**DOI:** 10.1371/journal.pone.0199557

**Published:** 2018-06-27

**Authors:** Lyriane Apolinário de Araújo, Adriano Augusto Peclat De Paula, Hellen da Silva Cintra de Paula, Jessica Enocêncio Porto Ramos, Brunna Rodrigues de Oliveira, Keila Patrícia Almeida De Carvalho, Rafael Alves Guimarães, Rita de Cássia Gonçalves de Alencar, Eliza Carla Barroso Duarte, Silvia Helena Rabelo Santos, Vera Aparecida Saddi, Megmar Aparecida dos Santos Carneiro

**Affiliations:** 1 Institute of Tropical Pathology and Public Health, Federal University of Goiás, Goiânia, Goiás, Brazil; 2 Department of Urological Oncology, Araujo Jorge Hospital, Goiânia, Goiás, Brazil; 3 Department of Biological and Biomedical Sciences, Pontifical Catholic University of Goiás, Goiânia, Goiás, Brazil; 4 Pathology Department, Araujo Jorge Hospital, Goiânia, Goiás, Brazil; 5 Department of Pathology, University of Brasília, Brasília, Federal District, Brazil; 6 Faculty of Pharmacy, Federal University of Goiás, Goiânia, Goiás, Brazil; Istituto Nazionale Tumori IRCCS Fondazione Pascale, ITALY

## Abstract

**Background:**

Penile carcinoma (PC) is a rare, highly mutilating disease, common in developing countries. The evolution of penile cancer includes at least two independent carcinogenic pathways, related or unrelated to HPV infection.

**Objectives:**

To estimate the prevalence, identify HPV genotypes, and correlate with clinicopathological data on penile cancer.

**Methods:**

A retrospective cohort study involving 183 patients with PC undergoing treatment in a referral hospital in Goiânia, Goiás, in Midwestern Brazil, from 2003 to 2015. Samples containing paraffin embedded tumor fragments were subjected to detection and genotyping by INNO-LiPA HPV. The clinicopathological variables were subjected to analysis with respect to HPV positivity and used prevalence ratio (PR), adjusted prevalence ratio (PRa) and 95% confidence interval (CI) as statistical measures.

**Results:**

The prevalence of HPV DNA in PC was 30.6% (95% CI: 24.4 to 37.6), high-risk HPV 24.9% (95% CI: 18.9 to 31.3), and 62.5% were HPV 16. There was a statistical association between the endpoints HPV infection and HPV high risk, and the variable tumor grade II-III (p = 0.025) (p = 0.040), respectively. There was no statistical difference in disease specific survival at 10 years between the HPV positive and negative patients (p = 0.143), and high and low risk HPV (p = 0.325).

**Conclusions:**

The prevalence of HPV infection was 30.6%, and 80.3% of the genotypes were identified as preventable by anti-HPV quadrivalent or nonavalent vaccine. HPV infections and high-risk HPV were not associated with penile carcinoma prognosis in this study.

## Introduction

Penile carcinoma (PC) is a rare and aggressive disease with high mutilating potential. The incidence in the United States and Western Europe is estimated at 0.4%, however, in Africa, Asia and South America the incidence is about 6.0% [1–3].

Brazil is a country with a high incidence of PC, accounting for about 2% of neoplasias that affect males, its frequency is associated with the studied region and socio-economic conditions of individuals [4–7].

The origin of penile carcinoma is multifactorial, and the incidence is mainly related to poor personal hygiene, high number of sexual partners, phimosis in adulthood and infections by bacteria and viruses, such as human papillomavirus (HPV) [2, 8–11]. Phimosis is a risk factor for this carcinoma, and circumcision is considered an important factor of prevention, and countries that adopt this practice have lower prevalence of PC [8,10,11].

Human papillomavirus (HPV) is the most common cause of sexually transmitted infection (STI) [12,13] and is considered an important etiologic agent for the development of PC, however, its role is not yet fully elucidated [14,15]. The development of penile carcinoma includes at least two independent carcinogenic routes, one being related to persistent HPV infection, and the other to no associated viruses, such as inflammatory conditions (chronic balanitis, lichen sclerosus), which are favored by the presence of phimosis [16–19]. HPV are classified according to their oncogenic potential, with approximately 15 types of high oncogenic risk involved in the carcinogenic process of some tumors through the action of viral oncoproteins (E6 and E7) [20–22].

The variations in the prevalence of HPV in PC, according to the literature, are due to differences in sampling, molecular testing, and study population [23]. The overall prevalence of HPV infection in penile neoplasia has been estimated from 13.4% to 55.6% worldwide [24]. In this multicenter study, the authors present HPV positivity rates by region: Europe (32.2%; 95% CI: 27.8 to 36.9), North America (18.8%; 95% CI: 4.0 to 45.6), Latin America (36.5%; 95% CI: 32.1 to 40.9), Africa (36.8%; 95% CI: 16.3 to 61.6), Asia (13.4%; 95% CI 6.3 to 24.0) and Oceania (55.6%; 95% CI: 21.2 to 86.3) [24]. In some histological types of PC persistent HPV infection is associated with genotype 16 [19].

In Brazil, studies carried out in patients with PC found HPV prevalence ranging between 30.5% and 63.1% in the states of São Paulo and Maranhão, respectively [25,26]. In 2011, an investigation conducted in Goiânia, capital of the State of Goiás in Midwestern Brazil, HPV positivity was found in 43.3% of cases, where 50.9% were HPV16 and 25.5% were HPV-18 [27].

Squamous carcinoma (SCC) is the most common histologic type of PC, representing about 95% of cases of this neoplasm. The HPV prevalence and penile carcinoma may differ between histologic types of squamous carcinoma [10,28,29]. Genotypic characterization of HPV in PC is important, in order to know the most frequent types. Giuliano and colleagues found that immunization with the quadrivalent vaccine resulted in 90.4% protection (95% CI: 45.8 to 98.1) against lesions related to HPV 6, 11, 16 and 18 in men [30], showing that the adoption of the HPV vaccine for men is a measure of prevention and control of this neoplasia.

The clinicopathological characteristics of penile tumors are factors that predict disease progression, the need for surgery, and death [15]. Therefore, this study aimed to estimate the prevalence and identify the HPV genotype and correlate these with clinicopathological data on penile carcinoma.

## Materials and methods

### Patients

This is a retrospective cohort study in patients with penile carcinoma treated in the Uro-Oncology service in a referral hospital in Goiânia, Goiás, Brazil, from January 2003 to November 2015. For this study, 225 patients received treatment during the defined period and were included in the study; of these, 42 were excluded, resulting in a total of 183 cases.

Inclusion criteria of patients in the study were: diagnosis with penile carcinoma and treatment in a referral hospital; biopsy or amputation of the penis in the institution; paraffin block with PC fragment and records located.

**Fig 1:**
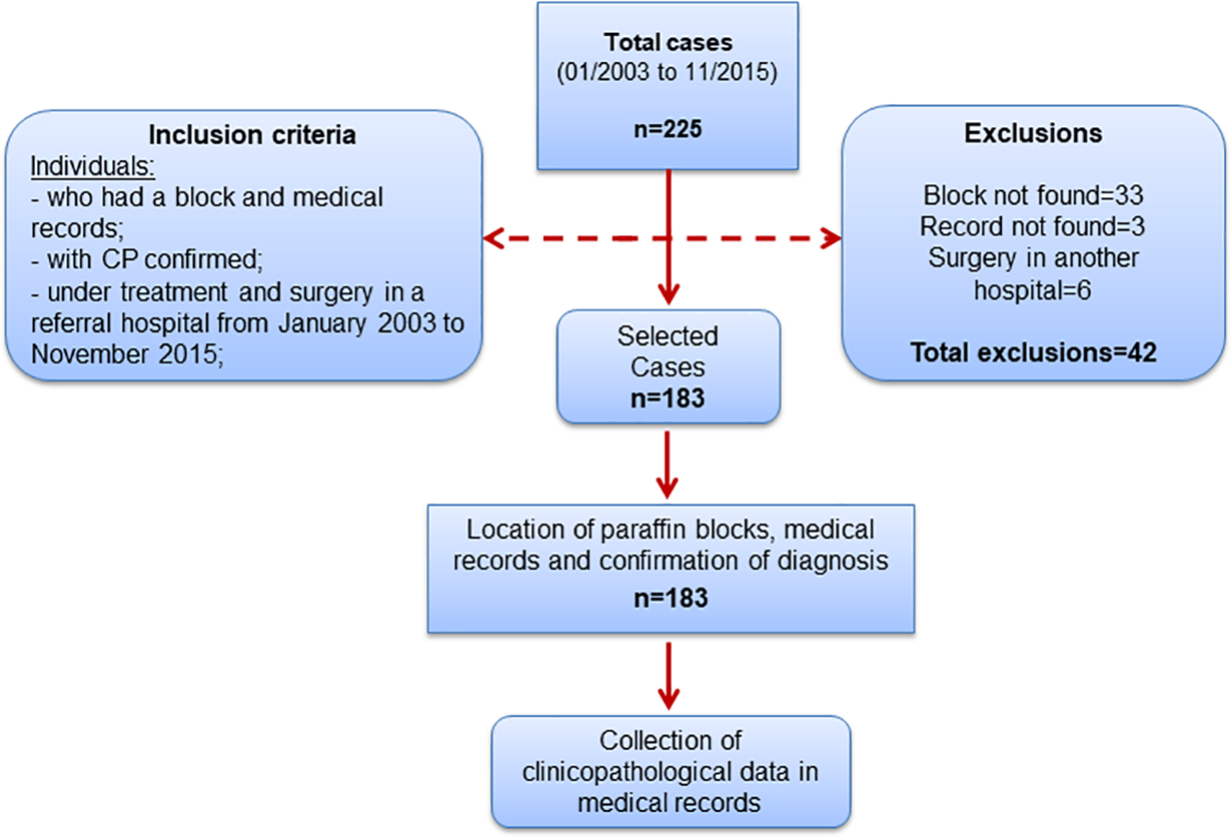
Flowchart of the study.

Cases whose paraffin blocks containing the fragment of the primary tumor were not found, and those submitted to neoadjuvant chemotherapy or penis surgery at another institution were excluded (Fig 1).

This study was approved by the Ethics Committee of the Association to Combat Cancer in Goiás (CEP / AACG) under a consolidated number CEP: 901.094.

### Preparation of samples

Slides containing paraffin processed tumor tissue fragments were stained with hematoxylin and eosin and evaluated by two pathologists independently to confirm the diagnosis of PC. After the selection of the cases, the blocks were cut into slices and stored in sterile 2 mL microtubes and identified by block number.

### Extraction, detection and genotyping of HPV DNA

The viral DNA extraction was performed with the following reagents: Xylol PA for removal of paraffin; Proteinase-K for cellular digestion; a commercial kit (Wizard Genomic DNA Purifications Kit—Promega) to precipitate protein: isopropanol for DNA precipitation and 70% ethanol for DNA purification.

The paraffin removal process results in loss of tissue, and therefore degradation of the DNA contained in the sample [31], so the integrity of DNA in samples for analysis were evaluated. Samples were subjected to polymerase chain reaction (PCR) using oligonucleotide primers specific for amplification of the enzyme glyceraldehyde-3-phosphate dehydrogenase (GAPDH) (human housekeeping gene) (INVITROGEN) (99 bp). GAPDH negative samples were re-extracted. Each amplification used a negative control (without DNA) and positive control.

Detection and genotyping the HPV DNA was accomplished using commercial kit HPV INNO-LiPA HPV genotyping extra (Fujirebio Europe, Ghent, Belgium) that amplifies the L1 viral region (65pb) using primer SPF 10. This method uses a primer which amplifies the human gene HLA-DPB1, used to monitor the quality of extraction of DNA from the sample. All reactions included negative control (without HPV-DNA) and positive control.

Genotyping was performed by reverse hybridization following amplification of the HPV L1 region, biotinylated amplicons were denatured and hybridized with specific probes fixed in parallel lines in strips. This method detected 28 genotypes, 15 genotypes of high-risk HPV, three probable high-risk, seven low risk, and three that were not classified according to risk. The tests were performed according to the manufacturer's instructions. To avoid cross-contamination between samples, specific laboratory work areas were designated for the handling of reagents and samples and for the manipulation of amplified products. Positive and negative controls were included in all DNA extractions and PCR amplification reactions. The protocols used for extraction, detection and genotyping of HPV DNA can be found in S1 Protocols.

### Statistical analysis

The dependent variables were: (i) HPV infection (no or yes) and (ii) High risk HPV infection (no or yes), and the following independent variables were analyzed: (i) age, categorized as < 60 years and ≥ 60 years; (ii) phimosis (no or yes); (iii) Jackson stage (0/II or III/IV); (iv) tumor grade (I or II/III); (v) tumor invasion (superficial, deep or in situ); (vi) inguinal metastasis (no or yes); (vii) inguinal lymphadenectomy (no or yes); (viii) inguinal recurrence (no or yes); (ix) lymphovascular invasion (absent or present) and (x) death (live or dead).

The variable phimosis was not included in regression analysis due to the large number of cases missing these variables in this study [32,33].

Data were analyzed in STATA, version 14.0. Initially, descriptive analysis was performed on all variables investigated. Quantitative variables were presented as mean and standard deviation (SD) and the qualitative variables as absolute and relative frequency. Factors associated with infection with HPV were made by Poisson regression with robust variance [34,35]. Variables with p values <0.10 of bivariate analyses were included in their respective models. Age, regardless of the p value, was included in the models due to confounding potential for the control. The results of the analyses are presented as prevalence ratio (PR), adjusted prevalence ratio (PRadj) and 95% CI. In addition, the log-rank test [36] was used to compare survival of PC patients between the following groups: (i) HPV+ versus and (ii) Low risk HPV and (ii) high-risk HPV. In all analyses, P values < 0.05 were considered statistically significant

## Results

A total of 183 individuals with penile carcinomas were included in the study. Fig 1 shows the algorithm of the study.

Most patients (51.4%) were 60 years or older at the time of diagnosis of PC. Phimosis was reported by (90.1%) of the participants. Tumor grade II-III was observed in 50.2% individuals, Jackson stage III-IV in 23.6% of cases, deep tumor invasion (51.9%), inguinal metastasis (36.1%), inguinal lymphadenectomy (38.8%) and post-surgical inguinal recurrence in 8.7% of patients. Regarding the primary treatment of lesions: partial penectomy 72.7%, total penectomy in 14.2% and emasculation in 4.4% of cases. The occurrence of death due to PC happened in 18.6% of the participants, according to records (Table 1). The study database can be found in S1 Database.

**Table 1:** Descriptive analysis of clinical variables in patients with penile carcinoma in Goiania, Goias, Brazil.

Variables	n	%	95% CI ^1^
**Age (years)**			
< 60	89	48.6	41.5–55.8
≥ 60	94	51.4	44.2–58.5
**Phimosis (n = 151)**			
No	15	9.9	6.1–15.7
Yes	136	90.1	84.3–93.9
**Tumor grade (n = 182)**			
I	90	49.5	43.3–56.6
II-III	92	50.5	43.4–57.7
**Jackson stage (N = 174)**			
0-II	133	76.4	69.6–82.1
III-IV	41	23.6	17.9–30.4
**Tumor invasion (n = 181)**			
Superficial	78	43.1	26.1–50.4
Deep	94	51.9	44.7–59.1
In situ	9	5.0	2.6–9.2
**Inguinal metastasis**			
No	117	63.9	56.8–70.5
Yes	66	36.1	39.5–43.2
**Inguinal lymphadenectomy**			
No	112	61.2	54.0–68.0
Yes	71	38.8	32.0–46.0
**Inguinal recurrence**			
No	167	91.3	86.3–94.5
Yes	16	8.7	5.4–13.7
**Tumor inflammatory infiltrate (n = 137)**			
Low	56	40.9	33.0–49.2
Moderate	67	48.9	40.7–57.2
Intense	14	10.2	6.2–16.4
**Lymphovascular invasion (n = 164)**			
Absent	137	83.5	68.1–50.6
Present	27	16.5	10.4–20.6
**Primary treatment**			
Partial penectomy	133	72.7	65.8–78.6
Total penectomy	26	14.2	9.9–20.0
Emasculation	8	4.4	2.2–8.4
Local Excision	16	8.7	5.4–13.7
**Death**			
Live	149	81.4	75.2–86.4
Dead	34	18.6	13.6–24.8

^1^95% confidence interval

The prevalence of HPV DNA was 30.6% in paraffin embedded tissue samples of PC. The high oncogenic risk genotypes were found in 24.9% of cases and low risk HPV in 3.8% (Table 2). Simple infection was found in 49 cases of HPV and multiple infection in seven cases (data not shown in table).

**Table 2:** Prevalence of HPV-DNA in 183 cases of penile carcinoma in a referral hospital in Goias, Brazil.

Variables	N = 183	%	95% CI ^1^
HPV+	56	30.6	24.4–37.6
HPV genotypes			
HPV high risk	45	24.9	18.9–31.3
HPV low risk	7	3.8	1.9–7.7
Undetermined*	4	2.2	0.9–5.5

^1^95% confidence interval

*four HPV positive samples did not have a genotype identified by LiPA, being called X

**Fig 2:**
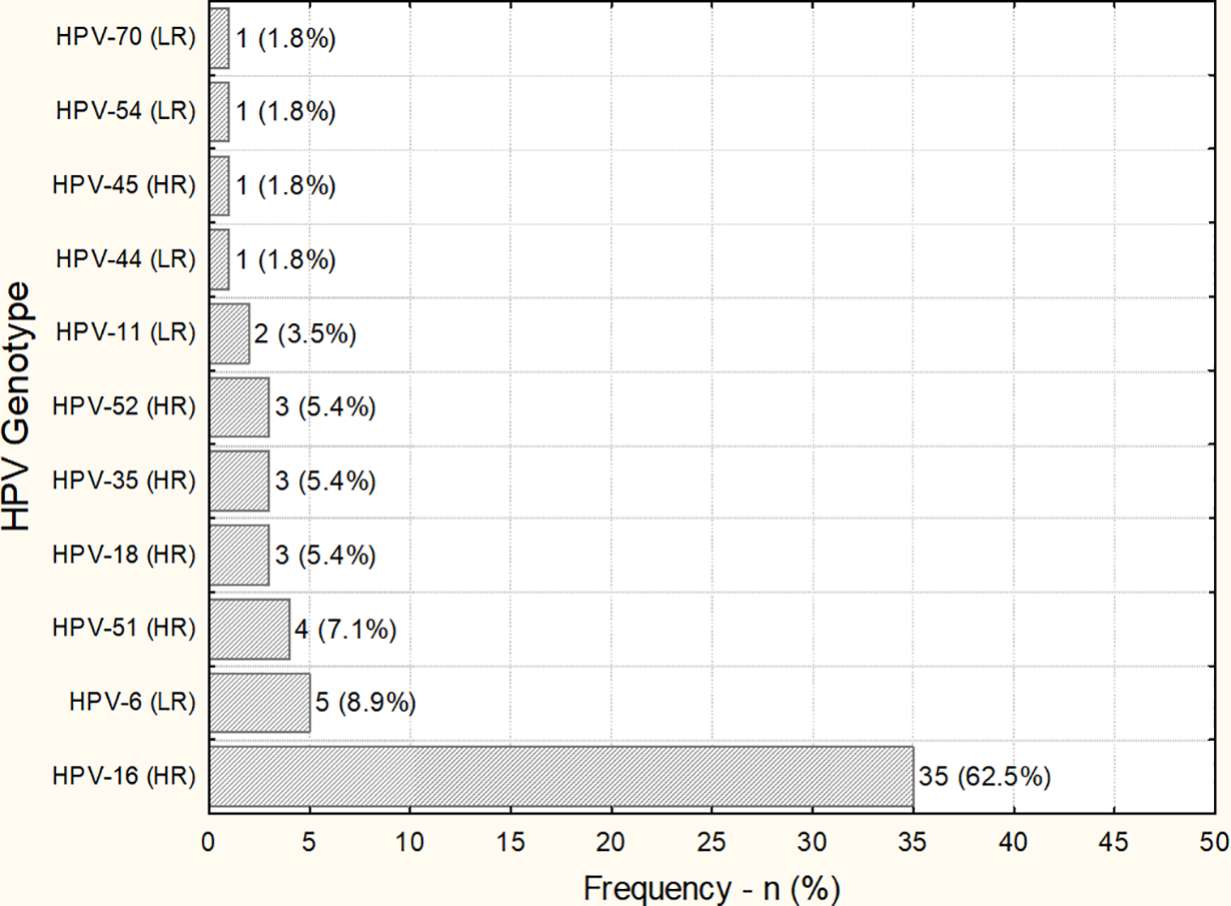
HPV genotype distribution in a population with PC. HR: High risk; LR: Low risk.

Among HPV DNA-positive samples, the most frequent HPV type was HPV 16 (62.5%) and HPV 18 (5.4%). HPV 6 and HPV 11 in total accounted for approximately 12.4% of penile carcinomas (Fig 2).

The clinical and pathological variables were submitted to bivariate analysis, with the outcome being the prevalence of HPV-DNA, only the variable tumor grade (II/III) remained associated after multivariate analysis (Table 3).

**Table 3:** Bivariate and multivariate analysis of factors associated with HPV infection.

Variables	Total (N = 183)	HPV+ (%)	PR (95% IC)	P^1^	Adjusted PR (95% IC)	p^1^
**Age (years)**						
< 60	89	28 (31.5)	1.00		1.00	
≥ 60	94	28 (29.8)	0.94 (0.61–1.46)	0.807	0.96 (0.61–1.47)	0.870
Phimosis*						
No	15	8 (53.3)	1.00			
Yes	136	37 (27.2)	0.51 (0.23–1.09)	0.084		
**Jackson stage**						
0-II	133	40 (30.1)	1.00			
III-IV	41	13 (31.7)	1.05 (0.62–1.77)	0.842		
**Tumor grade**						
I	90	20 (22.2)	1.00		1.00	
II-III	92	35 (38.0)	1.71 (1.07–2.73)	0.024	1.70 (1.06–2.72)	**0.025**
**Tumor invasion**						
Superficial	78	24 (30.8)	1.00			
Deep	94	29 (30.9)	1.00 (0.63–1.57)	0.991		
In situ	9	2 (22.2)	0.72 (0.20–2.57)	0.616		
**Inguinal metastasis**						
No	117	33 (28.2)	1.00			
Yes	66	23 (34.8)	1.23 (0.79–1.91)	0.346		
**Inguinal lymphadenectomy**						
No	112	32 (28.6)	1.00			
Yes	71	24 (33.8)	1.18 (0.76–1.83)	0.453		
**Inguinal recurrence**						
No	167	52 (31.1)	1.00			
Yes	16	4 (25.0)	0.80 (0.33–1.93)	0.625		
**Tumor inflammatory infiltrate**						
Low	56	13 (23.2)	1.00			
Moderate	67	19 (28.4)	1.22 (0.66–2.25)	0.521		
Intense	14	3 (21.4)	0.92 (0.30–2.81)	0.888		
**Lymphovascular invasion**						
Absent	137	40 (29.2)	1.00			
Present	27	9 (33.3)	1.41 (0.62–2.07)	0.663		
**Death**						
Live	149	51 (32.2)	1.00			
Dead	34	8 (23.5)	0.73 (0.28–1.40)	0.344		

Abbreviations: PR: prevalence ratio; 95% CI: 95% confidence interval

^1^Wald chi-square test

* Variable excluded from the multiple regression model due to the large amount of missing data.

The same variables were analyzed with the outcome as positive for high-risk HPV, age and tumor grade [II and III] included in the multivariate analysis model, remained statistically associated with the outcome the variable tumor grade II/III (Table 4).

**Table 4:** Bivariate and multivariate analysis of factors associated with infection by high-risk HPV.

Variables	Total (N = 179)	HPV High Risk Pos	PR (95% IC)	p^1^	Adjusted PR (95% IC)	p^1^
**Age (years)**						
< 60	87	21 (24.1)	1.00		1.00	
≥ 60	92	24 (26.1)	1.08 (0.64–1.79)	0.765	1.09 (0.66–1.81)	0.730
**Phimosis** ^*^						
No	14	5 (35.7)	1,00			
Yes	134	25 (18.7)	0.52 (0.19–1.36)	0.185		
**Jackson stage**						
0-II	130	32 (24.6)	1.00			
III-IV	40	10 (25.0)	1.01 (0.54–1.88)	0.961		
**Tumor grade**						
I	89	16 (18.0)	1.00		1.00	
II-III	89	28 (31.5)	1.75 (1.01–3.00)	0.043	1.76 (1.02–3.02)	**0.040**
**Tumor invasion**						
Superficial	76	20 (26.3)	1.00			
Deep	92	23 (25.0)	0.95 (0.56–1.59)	0.846		
In situ	9	1 (11.1)	0.42 (0.06–2.79)	0.372		
**Inguinal metastasis**						
No	115	27 (23.5)	1.00			
Yes	64	18 (28.1)	1.19 (0.71–2.00)	0.491		
**Inguinal lymphadenectomy**						
No	110	26 (23.6)	1.00			
Yes	69	19 (27.5)	1.16 (0.69–1.94)	0.558		
**Inguinal recurrence**						
No	163	42 (25.8)	1.00			
Yes	17	4 (23.5)	0.72 (0.25–2.09)	0.555		
**Tumor inflammatory infiltrate**						
Low	56	10 (17.9)	1.00			
Moderate	65	15 (23.1)	1.29 (0.62–2.65)	0.484		
Intense	14	3 (21.4)	1.20 (0.37–3.80)	0.757		
**Lymphovascular invasion**						
Absent	135	31 (23.0)	1.00			
Present	26	8 (30.8)	1.33 (0.69–2.58)	0.382		
**Death**						
Live	145	38 (26.2)	1.00			
Dead	34	7 (20.6)	0.78 (0.38–1.60)	0.509		

Abbreviations: PR: prevalence ratio; 95% CI: 95% confidence interval

^1^Wald chi-square test

* Variable excluded from the multiple regression model due to the large amount of missing data.

**Fig 3:**
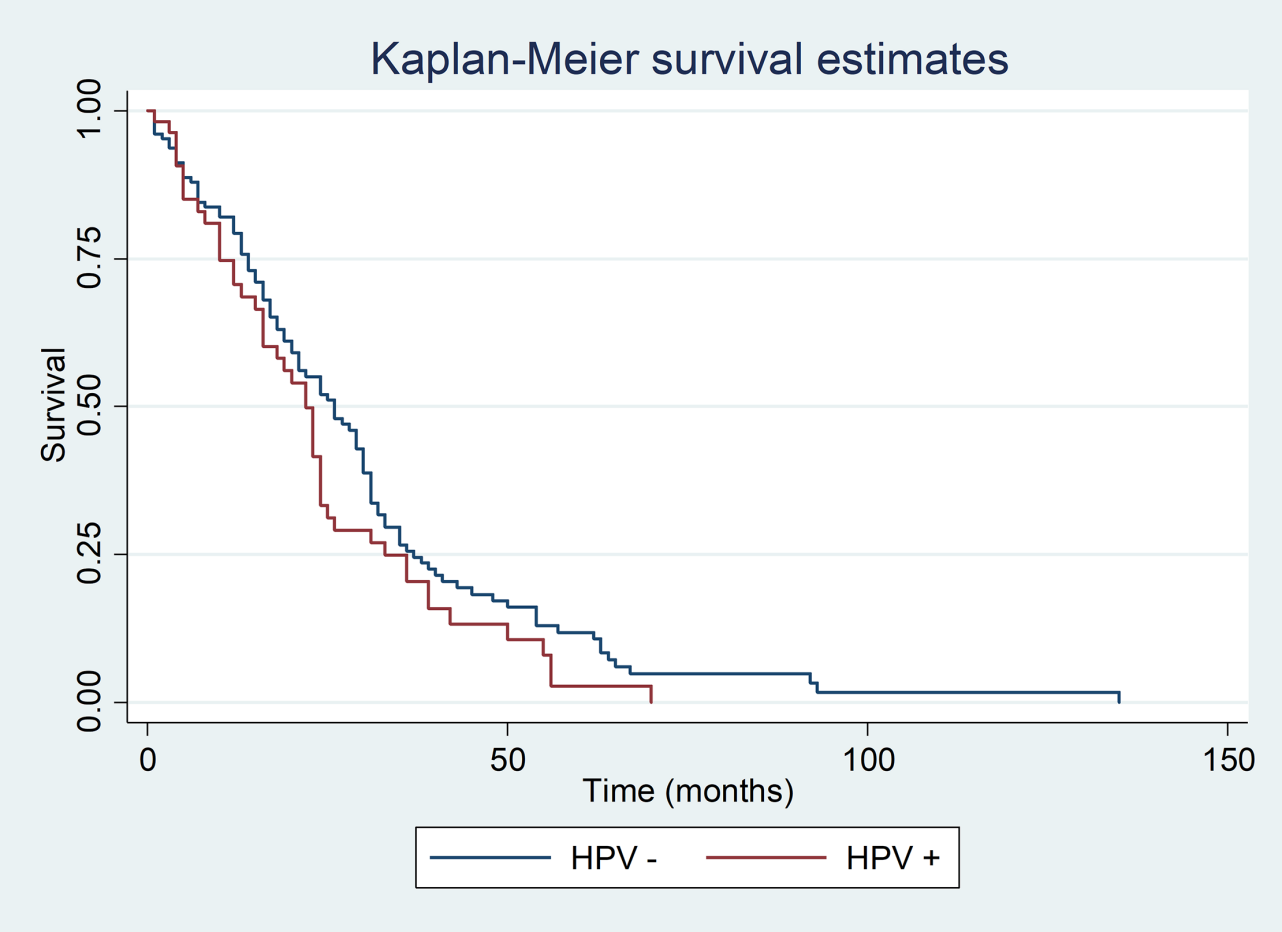
Curve of survival of patients positive and negative for HPV.

**Fig 4:**
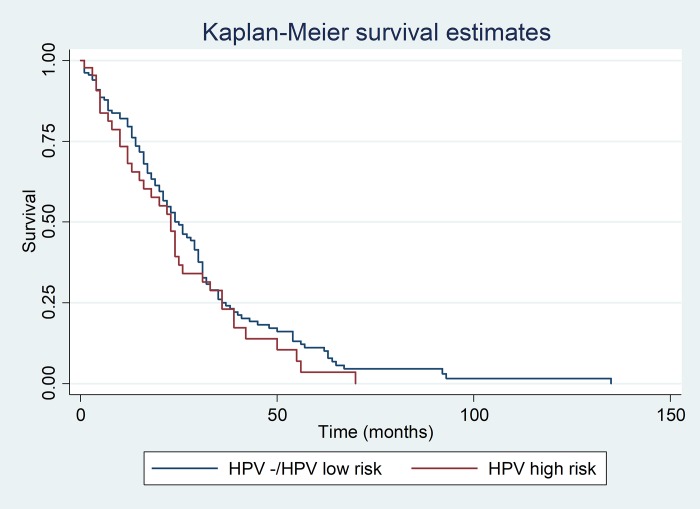
Survival curve of the patients in relation to HPV positivity and negativity low and high risk.

There was no statistical difference in survival between HPV positive and negative individuals over 10 years (long-rank test x^2^: 2.14, p = 0.143) (Fig 3). Regarding individuals infected with HPV high and low risk, no statistical difference with respect to survival (long-rank test x^2^: 0.97, p = 0.325) was found (Fig 4).

## Discussion

To our knowledge this is the largest number of cases of PC collected with the goal of estimating the prevalence, genotypic characterization, and HPV association with clinical and pathological characteristics of this tumor in Brazil.

Penile cancer occurs more frequently in men aged 50–70 years [37]. In fact, our results showed that most individuals were 60 years of age or older. This is consistent with other studies [11,38–40]. Early diagnosis is very important in relation to both the preservation of organs and the outcome of the disease, with survival rates estimated at approximately 50% over five years [37]. However, this condition also affects young individuals, in our study 13.1% of individuals with CP were between 24 and 40 years of age (data not shown).

In this study, presence of phimosis was observed in 90.1% of PC cases, corroborating literature data [9,26,41]. The increased risk of penile cancer among men with phimosis is associated with lichen sclerosis or inadequate penile hygiene, smegma retention and therefore infection. The meta-analysis showed that childhood circumcision may have a protective effect against penile cancer [42].

The pathogenesis of penile cancer is not well understood. A substantial percentage of penile carcinomas are associated with HPV while the remaining tumors rely on molecular mechanisms other than HPV [1,19].

The prevalence of HPV-DNA in penile carcinoma in this study was 30.6% (95% CI: 24.4–37.6). In a multicenter study conducted in 25 countries, the prevalence of HPV in PC was 33.1% (95% CI: 30.2–36.1) [24]. However, other studies conducted in the same population found a similarity in HPV positivity [24,40,43,44]. Other studies estimated higher rates of HPV positivity, being 46.9% (95% CI: 44.4–49.6) [45]; 47.8% (95% CI: 45.0–50.6) [23], 60.7% (95% CI: 51.9–69.0) [46] in paraffin-packed PC samples.

In Brazil, the prevalence of HPV in penile carcinoma ranges from 30.5 to 63.1% in paraffin-embedded samples [25–27,46,47,48]. This difference observed in HPV-DNA positivity may be related to the methodology of the studies, the selection of samples (paraffin or cryopreserved), incidence of HPV in the geographical regions, methods of viral detection, and population studied [14,16,24,43,49].

In this study, the prevalence of HPV and HPV types was estimated in 183 cases of PC, however, the research does not allow for inferences regarding viral activity, whether this viral infection is transcriptionally active or not. Other active infection markers would need to be investigated, for example, to evaluate expression of the p16^INK4a^ protein and detection of HPV E6*I mRNA [19,24]. However, this study is relevant from an epidemiological point of view and by virtue of the large number of PC cases included in the research.

The prevalence of high-risk oncogenic HPV genotypes in this study was 24.9% (45/183) and HPV-16 was identified in 62.5% of DNA-HPV positive samples, corroborating national and international data [15,18,24,26,47,50]. In a multicenter study, HPV 16 and 18 were detected in 70% of PC cases [24]. A similar finding was observed in this investigation, where 67.8% of the cases were infected with HPV 16 and 18.

HPV 6 and 11 are responsible for the development of 90% of genital warts [45] and have been identified in 12.5% of PC cases, similar to that found in other studies [26,48]. In our series, HPV vaccine types were detected in 80.3% CP cases. This study emphasizes the need for vaccine-related immunity in males to ensure a reduction in the overall burden of infection and diseases caused by HPV [30].

The role of HPV as a prognostic factor in penile cancer remains unclear [51]. It is uncertain whether cancers involving HPV infection have better survival profiles than cancers without HPV infection (15,40). In this series of cases, we observed no association between HPV and high-risk HPV negative and positive patients when considering lymph node metastasis, this being one of the primary factors related to patient survival.

In this study, infection by HPV and high-risk HPV was associated with tumor grade II / III (p = 0.025 / p = 0.040) in multivariate analysis, as well as in other studies [15,46,52]. This pathological variable indicates a worse prognosis of lesions [25], as the development of lymph node metastases increases according to the degree of cell indifferentiation [1].

A recent Netherlands study reported that High-risk HPV positive tumors appear to providence a significant survival benefit over High-risk HPV negative tumors on multivariable analsysis (hazard ratio [RH], 0.2; p = 0.034) [15]. However, these results differ from those observed in this study because there was no association between HPV (long-rank test x^2^: 2.14, p = 0.143) and high-risk and low-risk HPV (long-rank test x^2^: 0.97; p = 0.325) and disease-specific survival rate at 10 years. It is possible that these conflicting results may be due to different study designs, sample sizes and sampling methods for DNA-HPV (19,25).

This study has some limitations, by its own design. Because it was a retrospective investigation, there was no possibility of retrieving clinical and histopathological data not recorded in medical records. In addition, it was not possible to identify the histological subtypes of the cases of penile carcinoma and to correlate them with the prevalence of HPV, since the majority of anatomopathological reports of the tumors did not include the histological subtype. The study also did not investigate the transcriptional activity of HPV in PC, however, the study design was to estimate the prevalence of HPV and to correlate positivity with clinicopathological factors of PC cases.

## Conclusion

The results of the study showed that 80.3% of the types of HPV identified (16, 18, 6 and 11) in individuals with PC are immunopreventable types using the quadrivalent or nonavalent anti-HPV vaccine. This information emphasizes the importance of HPV vaccination in males, especially in developing countries, with a high incidence of penile carcinoma.

## Supporting information

S1 Database Study database. (XLSX)

S1 Protocols Protocols used for extraction, detection and genotyping of HPV DNA. (PDF)

## References

[1] Bleeker MC, Heideman DA, Snijders PJ, Horenblas S, Dillner J, Meijer C. Penile cancer: epidemiology, pathogenesis and prevention. World J Urol. 2009;27(2):141–50. doi: 10.1007/s00345-008-0302-z 18607597 10.1007/s00345-008-0302-z

[2] Morrison BF. Risk factors and prevalence of penile cancer. West Indian Med J. 2014 10;63(6):559–560. doi: 10.7727/wimj.2015.381 26225809 10.7727/wimj.2015.381 PMC4663967

[3] Spiess PE, Dhillon J, Baumgarten AS, Johnstone PA, Giuliano AR. Pathophysiological basis of human papillomavirus in penile cancer: Key to prevention and delivery of more effective therapies. CA Cancer J Clin. 2016 6 17; 66:481–95. doi: 10.3322/caac.21354 27314890 10.3322/caac.21354

[4] Costa S, Rodrigues R, Barbosa L, Silva JO, Brandão JOC, Medeiros CSQ. Câncer de pênis: Epidemiologia e estratégias de prevenção. Cadernos de graduação—Ciências biológicas e da saúde facipe. 2013;1(2):23–33. Portuguese.

[5] Favorito LA, Nardi AC, Ronalsa M, Zequi SC, Sampaio FJ, Glina S. Epidemiologic study on penile cancer in brazil. Int Braz J Urol. 2008 Sep-Oct;34(5):587–91. 18986562 10.1590/s1677-55382008000500007

[6] Instituto Nacional De Câncer [Internet]. Câncer De Pênis; 2016 [cited 2017 Nov 30]. Available from: http://www2.inca.gov.br/wps/wcm/connect/tiposdecancer/site/home/penis.

[7] BRASIL. Ministério Da Saúde. Política nacional de atenção integral à saúde do homem princípios e diretrizes. Série B. Textos Básicos De Saúde, Brasília-DF, 2009. Portuguese.

[8] Afonso LA, Cordeiro TI, Carestiato FN, Ornellas AA, Alves G, Cavalcanti SM. High risk human papillomavirus infection of the foreskin in asymptomatic men and patients with phimosis. J Urol. 2016 6;195(6):1784–9. doi: 10.1016/j.juro.2015.12.096 26796413 10.1016/j.juro.2015.12.096

[9] Beech B, Izawa J, Pautler S, Chin J, Power N. Penile cancer: Perspective from a Canadian tertiary care centre. Can Urol Assoc J. 2015;9(9–10):315–9. doi: 10.5489/cuaj.3230 26644802 10.5489/cuaj.3230 PMC4662391

[10] Christodoulidou M, Sahdev V, Houssein S, Muneer A. Epidemiology of penile cancer. Curr Probl Cancer. 2015 May-Jun;39(3):126–36. doi: 10.1016/j.currproblcancer.2015.03.010 26076979 10.1016/j.currproblcancer.2015.03.010

[11] Lobrigatte MFP, Rosolem SSM, Lobrigatte EOP, Rosolem WR, Saqueti EE. Clinical and epidemiological profile of penile cancer in regional referral service, Campo Mourão, Paraná. Brazilian Journal of Surgery and Clinical Research. 2015;9(1):20–3.

[12] World Health Organization [Internet]. Human papillomavirus (HPV) and cervical cancer. fact sheet 380. Jun 2016. [cited 2018 Jan 15]. Available from: http://www.who.int/mediacentre/factsheets/fs380/en/

[13] da Silva RJC, Sudenga SL, Sichero L, Baggio ML, Galan L, Cintra R, Torres BN, Stoler M, Giuliano AR, Villa LL. HPV-related external genital lesions among men residing in Brazil. Braz J Infect Dis. 2017 Jul-Aug;21(4):376–385. doi: 10.1016/j.bjid.2017.03.004 28399426 10.1016/j.bjid.2017.03.004 PMC6561086

[14] Scheiner MA, Campos MM, Ornellas AA, Chin EW, Ornellas MH, Andrada-Serpa MJ. Human papillomavirus and penile cancers in Rio de Janeiro, Brazil: HPV typing and clinical features. Int. Braz J Urol. 2008 8;34(4):467–76. doi: 10.1590/s1677-55382008000400009 18778498 10.1590/s1677-55382008000400009

[15] Djajadiningrat RS, Jordanova ES, Kroon BK, van Werkhoven E, de Jong J, Pronk DT, et al Human papillomavirus prevalence in invasive penile cancer and association with clinical outcome. Urol. 2015 2;193(2):526–31. doi: 10.1016/j.juro.2014.08.087 25150641 10.1016/j.juro.2014.08.087

[16] Diorio GJ, Giuliano AR. The role of human papilloma virus in penile carcinogenesis and preneoplastic lesions: a potential target for vaccination and treatment strategies. Urol Clin North Am. 2016 11;43(4):419–25. doi: 10.1016/j.ucl.2016.06.003 27717428 10.1016/j.ucl.2016.06.003

[17] Downes MR. Review of in situ and invasive penil esquamous cell carcinoma and associated non-neoplastic dermatological conditions. J Clin Pathol. 2015;68:333–40. doi: 10.1136/jclinpath-2015-202911 25883161 10.1136/jclinpath-2015-202911

[18] Mannweiler S, Sygulla S, Winter E, Regauer S. Two major pathways of penile carcinogenesis: HPV-induced penile cancers overexpress p16ink4a, HPV-negative cancers associated with dermatoses express p53, but lack p16ink4a overexpression. J Am Acad Dermatol. 2013 7;69(1):73–81. doi: 10.1016/j.jaad.2012.12.973 23474228 10.1016/j.jaad.2012.12.973

[19] Stratton KL, Culkin DJ. A contemporary review of HPV and penile cancer. Oncology (Williston Park). 2016 3;30(3):245–9. 26984219

[20] Bernard HU, Burk RD, Chen Z, van Doorslaer K, zur Hausen H, de Villiers EM. Classification of papillomaviruses (PVs) based on 189 PV types and proposal of taxonomic amendments. Virology. 2010 5;401(1):70–79. doi: 10.1016/j.virol.2010.02.002 20206957 10.1016/j.virol.2010.02.002 PMC3400342

[21] García-Espinosa B, Nieto-Bona MP, Rueda S, Silva-Sánchez LF, Piernas-Morales MC, Carro-Campos P, et al Genotype distribution of cervical human papillomavirus DNA in women with cervical lesions in Bioko, Equatorial Guinea. Diagn Pathol. 2009 9 9;4:31 doi: 10.1186/1746-1596-4-31 19740435 10.1186/1746-1596-4-31 PMC2749013

[22] Woodman CB, Collins SI, Young LS. The natural history of cervical HPV infection: unresolved issues. Nat Rev Cancer. 2007 1;7(1):11–22. doi: 10.1038/nrc2050 17186016 10.1038/nrc2050

[23] Backes DM, Kurman RJ, Pimenta JM, Smith JS. Systematic review of human papillomavirus prevalence in invasive penile cancer. Cancer Causes Control. 2009 5;20(4):449–57. doi: 10.1007/s10552-008-9276-9 19082746 10.1007/s10552-008-9276-9

[24] Alemany L, Cubilla A, Halec G, Kasamatsu E, Quirós B, Masferrer E, et al; HPV VVAP Study Group. Role of human papillomavirus in penile carcinomas worldwide. Eur Urol. 2016 5;69(5):953–61. doi: 10.1016/j.eururo.2015.12.007 26762611 10.1016/j.eururo.2015.12.007

[25] Bezerra AL, Lopes A, Santiago GH, Ribeiro KC, Latorre MR, Villa LL. Human papillomavirus as a prognostic factor in carcinoma of the penis: analysis of 82 patients treated with amputation and bilateral lymphadenectomy. Cancer. 2001 6 15;91(12):2315–21. 11413520

[26] de Sousa ID, Vidal FC, Branco Vidal JP, de Mello GC, do Desterro Soares Brandão Nascimento M, Brito LM. Prevalence of human papillomavirus in penile malignant tumors: Viral genotyping and clinical aspects. BMC Urol. 2015 2 24;15:13 doi: 10.1186/s12894-015-0007-8 25887354 10.1186/s12894-015-0007-8 PMC4349728

[27] de Paula AA, Motta ED, Alencar RC, Saddi VA, da Silva RC, Caixeta GN, et al The impact of cyclooxygenase-2 and vascular endothelial growth factor C immunoexpression on the prognosis of penile carcinoma. J Urol. 2012 1;187(1):134–40. doi: 10.1016/j.juro.2011.09.027 22088344 10.1016/j.juro.2011.09.027

[28] Cubilla AL, Velazquez EF, Young RH. Epithelial lesions associated with invasive penile squamous cell carcinoma: a pathologic study of 288 cases. Int J Surg Pathol. 2004 10;12(4):351–64. doi: 10.1177/106689690401200408 15494861 10.1177/106689690401200408

[29] Moch H, Cubilla AL, Humphrey PA, Reuter VE, Ulbright TM. The 2016 WHO classification of tumours of the urinary system and male genital organs-part a: Renal, penile, and testicular tumours. Eur Urol. 2016 7;70(1):93–105. doi: 10.1016/j.eururo.2016.02.029 26935559 10.1016/j.eururo.2016.02.029

[30] Giuliano AR, Palefsky JM, Goldstone S, Moreira ED Jr, Penny ME, Aranda C, et al Efficacy of quadrivalent HPV vaccine against HPV infection and disease in males. N Engl J Med. 2011 2 3;364(5):401–11. doi: 10.1056/NEJMoa0909537 21288094 10.1056/NEJMoa0909537 PMC3495065

[31] Steinau M, Patel SS, Unger ER. Efficient DNA extraction for HPV genotyping in formalin-fixed, paraffin-embedded tissues. J Mol Diagn. 2011 7;13(4):377–81. doi: 10.1016/j.jmoldx.2011.03.007 21704270 10.1016/j.jmoldx.2011.03.007 PMC3123789

[32] Langkamp DL, Lehman A, Lemeshow S. Techniques for Handling Missing Data in Secondary Analyses of Large Surveys. Acad Pediatr. 2010 May-Jun;10(3):205–10. doi: 10.1016/j.acap.2010.01.005 20338836 10.1016/j.acap.2010.01.005 PMC2866831

[33] Rubin LH, Witkiewitz K, Andre JS, Reilly S. Methods for Handling Missing Data in the Behavioral Neurosciences: Don’t Throw the Baby Rat out with the Bath Water. 6 2007,5(2):A71–A77. PMC3592650 23493038

[34] Coutinho LM, Scazufca M, Menezes PR. methods for estimating prevalence ratios in cross-sectional studies. Rev Saude Publica. 2008 12;42(6):992–8. 19009156

[35] Barros AJ, Hirakata VN. Alternatives for logistic regression in cross-sectional studies: An empirical comparison of models that directly estimate the prevalence ratio. BMC Med Res Methodol. 2003 10 20;3:21 doi: 10.1186/1471-2288-3-21 14567763 10.1186/1471-2288-3-21 PMC521200

[36] Bland JM, Altman DG. The logrank test. BMJ. 2004 5 1;328(7447):1073 doi: 10.1136/bmj.328.7447.1073 15117797 10.1136/bmj.328.7447.1073 PMC403858

[37] Pow-Sang MR, Ferreira U, Pow-Sang JM, Nardi AC, Destefano V. Epidemiology and natural history of penile cancer. Urology. 2010 8;76(2 Suppl 1):S2–6. doi: 10.1016/j.urology.2010.03.003 Review. 20691882 10.1016/j.urology.2010.03.003

[38] Bezerra SM, Chaux A, Ball MW, Faraj SF, Munari E, Gonzalez-Roibon N, et al human papillomavirus infection and immunohistochemical p16ink4a expression as predictors of outcome in penile squamous cell carcinomas. Human Pathology. 2015 4;46(4):532–40. doi: 10.1016/j.humpath.2014.12.004 25661481 10.1016/j.humpath.2014.12.004

[39] López-Romero R, Iglesias-Chiesa C, Alatorre B, Vázquez K, Piña-Sánchez P, Alvarado I, et al HPV frequency in penile carcinoma of mexican patients: Important contribution of HPV16 european variant. Int J Clin Exp Pathol. 2013; 6(7):1409–15. 23826423 PMC3693207

[40] Steinestel J, Ghazal AA, Arndt A, Schnoeller TJ, Schrader AJ, Moeller P, et al The role of histologic subtype, p16ink4a expression, and presence of human papillomavirus DNA in penile squamous cell carcinoma. BMC Cancer. 2015 4;15:220 doi: 10.1186/s12885-015-1268-z 25885064 10.1186/s12885-015-1268-z PMC4392470

[41] Bozzini G, Provenzano M, Romero Otero J, Margreiter M, Garcia Cruz E, Osmolorskij B, et al Role of penile doppler us in the preoperative assessment of penil esquamous cell carcinoma patients: results from a large prospective multicenter european study. Urology. 2016 4;90:131–35. doi: 10.1016/j.urology.2016.01.003 26776562 10.1016/j.urology.2016.01.003

[42] Larke NL, Thomas SL, dos Santos Silva I, Weiss HA. Male circumcision and penile cancer: a systematic review and meta-analysis. Cancer Causes Control 2011;22:1097–110. doi: 10.1007/s10552-011-9785-9 21695385 10.1007/s10552-011-9785-9 PMC3139859

[43] Cubilla AL, Lloveras B, Alejo M, Clavero O, Chaux A, Kasamatsu E, et al The basaloid cell is the best tissue marker for human papillomavirus in invasive penile squamous cell carcinoma: A study of 202 cases from Paraguay. Am J Surg Pathol. 2010 1;34(1):104–14. doi: 10.1097/PAS.0b013e3181c76a49 20035150 10.1097/PAS.0b013e3181c76a49

[44] Barzon L, Cappellesso R, Peta E, Militello V, Sinigaglia A, Fassan M, et al Profiling of expression of human papillomavirus-related cancer mirnas in penile squamous cell carcinomas. Am J Pathol. 2014 12;184(12):3376–83. doi: 10.1016/j.ajpath.2014.08.004 25455689 10.1016/j.ajpath.2014.08.004

[45] Miralles-Guri C, Bruni L, Cubilla AL, Castellsagué X, Bosch FX, de Sanjosé S. Human papillomavirus prevalence and type distribution in penile carcinoma. J Clin Pathol. 2009 10;62(10):870–8. doi: 10.1136/jcp.2008.063149 19706632 10.1136/jcp.2008.063149

[46] Afonso LA, Moyses N, Alves G, Ornellas AA, Passos MR, Oliveira LH, et al Prevalence of human papillomavirus and epstein-barr virus DNA in penile cancer cases from Brazil. Mem Inst Oswaldo Cruz. 2012 2;107(1):18–23. 22310531 10.1590/s0074-02762012000100003

[47] Calmon MF, Mota MTO, Babeto E, Candido NM, Girol AP, Mendiburu CF, et al Overexpression of ANXA1 in penile carcinomas positive for high-risk HPVs. Plos One. 2013;8(1):e53260 doi: 10.1371/journal.pone.0053260 23341933 10.1371/journal.pone.0053260 PMC3544802

[48] Fonseca AG, Soares FA, Burbano RR, Silvestre RV, Pinto LOAD. Human papillomavirus: Prevalence, distribution and predictive value to lymphatic metastasis in penile carcinoma. Int Braz J Urol. 2013 Jul-Aug;39(4):542–50. doi: 10.1590/S1677-5538.IBJU.2013.04.12 24054382 10.1590/S1677-5538.IBJU.2013.04.12

[49] Ferrandiz-Pulido C, Masferrer E, Torres I, Lloveras B, Hernandez-Losa J, Mojal S, et al Identification and genotyping of human papillomavirus in a spanish cohort of penile squamous cell carcinomas: Correlation with pathologic subtypes, p16ink4a expression, and prognosis. J Am Acad Dermatol. 2013 1;68(1):73–82. doi: 10.1016/j.jaad.2012.05.029 22863066 10.1016/j.jaad.2012.05.029

[50] Hernandez BY, Goodman MT, Unger ER, Steinau M, Powers A, Lynch CF, et al; HPV Typing Of Cancer Workgroup. Human papillomavirus genotype prevalence in invasive penile cancers from a registry-based United States population. Front Oncol. 2014 2 5;4:9 doi: 10.3389/fonc.2014.00009 24551592 10.3389/fonc.2014.00009 PMC3914298

[51] Kidd LC, Chaing S, Chipollini J, Giuliano AR, Spiess PE, Sharma P. Relationship between human papillomavirus and penile cancer-implications for prevention and treatment. Transl Androl Urol. 2017 10;6(5):791–802. doi: 10.21037/tau.2017.06.27 29184775 10.21037/tau.2017.06.27 PMC5673821

[52] Lucie GAL, Cubilla VE, Reuter GP, Haas WD. Preferential association of human papillomavirus with high-grade histologic variants of penile-invasive squamous cell carcinoma. Journal of The National Cancer Institute. 1995 11; 87(22):1705–09. doi: 10.1093/jnci/87.22.1705 7473819 10.1093/jnci/87.22.1705

