# Supplementary material for: Human papillomavirus (HPV) genotype distribution in penile carcinoma: Association with clinic pathological factors
Source: PLoS One. 2018 Jun 27;13(6):e0199557. doi: 10.1371/journal.pone.0199557 (PMC6021089; doi:10.1371/journal.pone.0199557)
Supplement: S1 Protocols — (PDF) [file pone.0199557.s002.pdf]

Standard Operating Procedure: EXTRACTION OF DNA FROM MATERIAL ENCASED IN PARAFIN USING XYLOL

1. OBJECTIVE: This procedure describes the extraction of DNA drom materials encased in parafin blocks using an organic solvent for removal of the parafin, digestion with Proteinase-K, precipitation of proteins with a Promega Kit, precipitation of DNA with 70% Isopropynol and Ethanol for the purification of DNA.

2. MATERIALS AND METHODS:

2.1 Equipment:

- Thermoblock;
- Cooler/Refrigerator;
- Freezer -80 degrees celsius (°C);
- Centrifuge without refrigeration (range to 13.000 rotations per minute (rpm);

2.2 Materials:

- Polypropylene microtubes 1.5 milliliters (ml);
- Micropipettes, 20, 200 and 1000 microliters (µl) and respective tips;
- Absorbent Paper;
- Source;

2.3 Reagents for the extraction of DNA:

- Deionized warer.
- MiliQ Autoclaved Water.
- Promega Kit: Cellular Lysis Solution and precipitation of proteins
- Proteinase-K 2.0% (100 milligrams (mg) Proteinase K and 5 ml TE pH 9).
- 100% Ethanol: Hold aliquots in sterile falcon tube at -4°C.
- Ethanol 70%: Hold aliquots in sterile falcon tube with 70 ml of 100% ethanol and 30 ml of deionized water and store at -4°C. Prepare with sterile Milli-Q water and sterile beaker.
- Xylol: Hold aliquots in sterile falcon tube at -4°C.
- Isopropanol: Hold aliquots in sterile falcon tube at -4°C.

2.4 Disinfection of work surfaces, laminar flow and accessories:

- Laminar flow, benches, micropipettes, microcentrifuge should be disinfected with sodium hypochlorite 1.0%.

2.5 Personal Protective Equipment (PPE):

- Glove and Apron - To be used throughout the procedure, noting that for the handling of Polymerase Chain Reaction (PCR) reagents it is recommended to use gloves and aprons that have not been previously exposed to the amplified product. Do not enter the PCR preparation room or the DNA extraction room with an apron exposed to amplified product.

- Mask - Use while handling Xylol. Work under a fume hood or in a place with sufficient exhaust.

3. PROCEDURE
3.1 DNA Extraction

<u>1st Step:</u>

Deparafinizing Samples
1. Place the material slice in a 1.5ml tube;
2. Add 500 µL de xylol;
3. Incubate for 15 minutes at 65°C in a thermoblock;
4. Centrifuge at 13000 rpm for 5 minutes to form a pellet of material;
5. Drain xylol using a pipette;
6. Repeat steps 2, 3, 4 and 5 twice more to complete the parafin removal process;
7. Add 500 µL of 100% ethanol (at room temperature) to all samples;
8. Incubate the samples for 15 minutes at 65°C in a thermoblock;
9. Centrifuge at 13000 rpm for 5 minutes to form a pellet;
10. Drain the ethanol using a pipette;
11. Repeat steps 7, 8, 9 and 10 two additional times to complete the removal of the paraffin.

Note: At this point, remove Proteinase-K 2% from the freezer.

Cellular Lysis
1. Add 200 µL of cell lysis solution, then macerate the entire sample with the tip;
2. Add 12 µL of 2% Proteinase-K to the sample;
3. Homogenize the reaction product with a pipette and incubate at 65°C in a thermoblock until complete cell digestion is achieved (Overnight).

Note: Some samples require longer time and higher Proteinase-K concentrations to complete cell digestion. If there is paraffin in the sample or the sample remains very opaque after incubation overnight, pipette another 5 µL of 2% Proteinase-K and incubate for another 30 minutes in a thermoblock.

<u>2nd Step:</u>

Precipitation of Proteins
1. Cool the samples at room temperature for 5 minutes;
2. Add 50 µL of protein precipitation solution to the cell lysate and homogenize with pipette;
3. Incubate at -20°C for 15 minutes;

Note: Set up new tubes to hold the sample in a later step.

4. Centrifuge at 13000 rpm for 15 minutes;

Note: The protein precipitate will form a pellet at the bottom of the tube. If the precipitate is not visible, repeat steps 2 and 4.

Precipitation of DNA

1. Transfer the supernatant with the DNA to a 1.5 ml autoclaved microtube using a pipette;
2. Add 200 µL of 100% isopropanol (stored in a refrigerator, remove it at the time of use);
3. Homogenize well by inversion;
4. Incubate for one hour in a freezer at -80°C;
5. Centrifuge at 13000 rpm for 5 minutes;
6. Discard isopropanol by inversion;
7. Dry the inverted tube on clean absorbent paper for 15 minutes;
8. Add 200 µl of 70% ethanol (stored in a freezer) and homogenize by inversion repeatedly to wash the DNA pellet;
9. Centrifuge the samples at 13,000 rpm for 5 minutes;
10. Discard the ethanol by inversion;
11. Dry the inverted tube on clean absorbent paper for 15 minutes.

Note 1: Remove ultrapure water from the freezer at this time; Note 2: Turn on ice machine at thie time.

Hydration of DNA

1. Add 20 µL of MiliQ Autoclaved Water;
2. Store at -20°C.

BIBLIOGRAPHIC REFERENCES:

SAMBROOK, J.; FRITSCH, E.F.; MANIATIS, T. Molecular cloning: a laboratory manual. 2nd ed., New York: Cold Spring Harbor Laboratory Press, 1989.

SANGUINETTI, C.J.; DIAS NETO, E.; SIMPSON, A.J.G. Rapid silver staining and recovery of PCR products separated on polyacrylamide gels. Biotechniques, 17: 915-9, 1994.

STANDARD OPERATING PROCEDURE: GENOTYPING OF HBV USING INNO-LiPA HPV Genotyping Extra Kit

1. OBJECTIVE: This procedure aims to identify 28 different genotypes of human papilomavirus (HPV) through the specific detection of sequences of the L1 genome region of HPV using the *USING INNO-LiPA HPV Genotyping Extra Kit* and Autoblot 3000H automated system.

2. RESPONSIBILITY: All employees of the Department must be trained in this procedure.

3. DEFINITIONS:
PPE: Personal Protective Equipment

PCR: Polymerase Chain Reaction

4. MATERIALS AND METHODS:

4.1 - Kit for Amplification and Genotyping

4.1.1 – Amplification Kit

The amplification kit has 3 components: the mixture for the PCR (AMP MIX), the enzyme (ENZ MIX), and a positive control for the reaction (CONTROL+) that are transported and kept at -20°C.

4.1.2 – Genotyping Kit

The genotyping Kit consists of the following items:
- STRIPS -20 genotyping strips;
- DENATURATION SOLUTION - Alkaline solution containing EDTA for sample denaturation;
- HIBRIDIZATION SOLUTION - SSC buffer containing SLS - sodium lauryl sulfate;
- STRINGENT WASH SOLUTION - SSC buffer containing SLS;
- CONJUGATE 100X – Solution of phosphatase-labeled streptoavidin solution in a TRis buffer containing stabilized protein and preservative. This should be diluted 1/100 with conjugate diluent
- CONJUGATE DILUENT- Phosphate buffer containing MaCl Triton stabilizing proteins, and preservative;
- SUBSTRATE BCIP/NBT 100x - BCIP and NBT in DMF to be diluted 1/100 in substrate buffer;
- SUBSTRATE BUFFER - Solution containing Tris, NaCl, MgCl2 buffer and preservatives;
- RINSE SOLUTION 5X- Phosphate buffer containing NaCl, Triton, and preservatives to be diluted in deionized water 1:5.

4.2 Equipamentos:

- Thermocycler;
- Micropipettes 20, 200, and 1000 µl and respective tips;
- Autoblot 3000H;
- 200 ml and 50 ml tubes;
- 2 beakers;
- Plastic tray with 20 channels (not included with KIT).

4.3 – Reagents

- Deionized Water;
- INNO-LiPA HPV Genotyping Extra;
- INNO-LiPA HPV Genotyping Extra Amp;

Solution of sodium hypochlorite diluted 1:10 in distilled water.

4.4 – Disinfection of surfaces, laminar flow, and accessories
Disinfection of laminar flow, benches and micropipettes shall be performed before and after the procedure with hypochlorite diluted 1:10 with deionized water.

4.5 - PPE
- Gloves throughout the procedure;
- Surgical mask;
- 2 aprons, one for the preparation of the samples and another for handling the amplified product during genotyping.

5. PROCEDURE

5.1 *Preparing the Reaction*

1- Prepare the Master Mix according to the number of samples, including the positive and negative controls and an extra tube. Homogenize and vortex the tubes before use.
2- For one (1) sample: Pipette 37.7 µl of AMP MIX and 2.3 µl of ENZ MIX. Dispense 40 µl of the mixture into each tube and add 10 µl of the DNA solution to achieve a final concentration of 100ng/µl.
3- At the end of the reaction process the samples immediately or keep them at -20ºC +/- 5ºC until ready to use.

PCR Cycles

1- 37ºC for 10 minutes
2- 94ºC for 9 minutes
3- 94ºC for 30 sec
4- 52ºC for 45 sec
5- 72ºC for 45sec
6- Repeat Steps 3-5 40 times
7- 72ºC for 30 seconds

5.2 Genotyping

Hybridization and stringent wash incubations should be performed at exactly **49°C +/- 0.5°C** and are the most critical steps to avoid a false-positive (very low temperature) or false-negative / very weak signals (very high temperature). For this reason, the equipment must be initialized prior to heating both solutions.

Turn on the Autoblot-300H and recall the saved program, follow the instructions in the program to proceed through the test step by step.

While the solutions are heating (about 30 minutes), prepare the solutions described below, set up the tray and the strips, and 10 minutes before the end of the heating period, prepare for product denaturation as below.

5.2.1 Denaturization of Amplified Product

- Pipette 10 µl of the denaturation solution and 10 µl of the amplified product into the upper corner of the tray, taking care not to open the tube over the tray. Carefully homogenize the mixture with the tip of the pipette itself.
- Keep the mixture at room temperature in contact for 10 minutes (this time can be extended up to 15 minutes)
- Five minutes before the end of the denaturing time, place the already identified strips inside the tray. SEE ITEM 5.2.2. (D) describing the position of the strips.

5.2.2 Dilution of genotyping solution:

A – Dilution of conjugate

The working solution should be diluted 1/100 with the **conjugate diluent**. Add two ml per channel. For volume calculation, use the volume the number of channels plus two ml over. Once prepared, the conjugate solution will be stable for 8 hours at room temperature (20-25ºC).

n	Conjugate (ml)	Diluent (ml)	Last (ml)
24	0,50	49,5	50
20	0,42	41,58	42
12	0,26	25,74	26
10	0,21	21,79	22
9	0,2	19,8	20
8	0,18	17,82	18
7	0,16	15,84	16

B- Dilution of substrate

The working solution should be diluted 1/100 in **substrate buffer** prior to use. Add two ml of substrate per channel and calculate two ml over. The diluted substrate solution is stable for 8 hours at room temperature (20-25°C) if kept away from light

n	Substrate (ml)	Substrate buffer (ml)	Last (ml)
24	0,50	49,5	50
20	0,42	41,58	42
12	0,26	25,74	26
10	0,21	21,79	22
9	0,2	19,8	20
8	0,18	17,82	18
7	0,16	15,84	16

C- Rinse Solution 5X: The wash solution should be diluted 1/5 **in distilled water**. Prepare eight ml of washing solution for each strip and another 10 ml over. The diluted wash solution is stable for two weeks at 2-8°C.

n	Rinse Solution 5X (ml)	Water (ml)	Last (ml)
24	40,4	161,6	202
20	34	136	170
12	21,2	84,8	106
10	18	72	90
9	16,4	65,6	82
8	14,8	59,2	74
7	13,2	52,8	66

D- PLACEMENT OF STRIPS ON TRAY

- Strips 1-10 should be placed starting from the right of the center channel.
- Strips 11-20 should be placed in the channels starting from the left of the center channel.
- During the experiment the equipment cover should remain closed.

20	19	18	17	16	15	14	13	12	11	NO STRIP	1	2	3	4	5	6	7	8	9	10

TO PROCEED WITH FEWER THAN 10 STRIPS:

When the test has less than 10 strips, calculate the volume of the stringent hybridization and wash solutions considering three additional channels: the center channel, the left channel of the center, and one channel to the right of the last channel with a strip (Y=N+3).

TO PROCEED WITH FEWER THAN 10 STRIPS AND LESS THAN 20:

When the assay has 10 or more than 10 strips but less than 20, calculate the volume of the stringent hybridization and wash solutions by considering two additional channels: the center channel, and one to the left of the last strip or to the left of the center channel (Y=N+2).

TO PROCEED WITH MORE THAN 20 STRIPS:

When the test has 20 channels filled, use an additional channel, in this case use the center channel for the calculation of the volume of the hybridization solution and stringent washing solution (Y=N+1).

1- Identify the channels at the top of the tray and place the required number of strips into the reservoirs as outlined above.
2- Place **10 µl of denaturation solution** in the upper corner of the reservoir. Close the bottle immediately after use.
3- Put **10 µl of the amplified product** into the drop of the denaturation solution and mix it with the tip of the pipette itself several times. Leave for **five minutes at room temperature** (solution can remain up to 15 minutes).
4- Five minutes before the end of the denaturation, **place the strips** in the channels of the tray.
5- Place the tray inside the machine and follow the required steps.

5.3 HOW TO ADD A NEW PROGRAM TO THE AUTOBLOT 3000H:
The program for the development of the strips contains **nove etapas**.
After the self-initialization of the Autoblot 3000H the following prompt will appear:
"READY FOR A NEW TEST?" press "**NO"** to begin a new program.
.
Press the ↑ **or** ↓ arrows to select an "**EMPTY**" program and press **"ENTER".**
Next, Enter the programming steps where the conditions of temperature, incubation, dispensed volumes, stirring speed, number of cycles, and aspiration after each incubation.
The following is the program for the genotyping assay using the INNO-LiPA HPV Extra Kit, Fujirebio, saved as **LIPAS.**

1- STEP 1= HYBRIDIZATION SOLUTION (HIBR)
Temperature= 49°C / Time= 1hora / Volume = 2 ml / Agitation= MED / **Cycle=1** / Aspiration= YES
2- STEP 2= STRINGENT WASH SOLUTION (STRIN)
Temperature= 49°C / Time= 1MIN / Volume = 2 ml / Agitation= MED / **Cycle=2** / Aspiration= YES
3- STEP 3= STRINGENT WASH SOLUTION (STRIN)
Temperature= 49°C / Time= 30min / Volume = 2 ml / Agitation= MED / **Cycle=1** / Aspiration= YES
4- STEP 4= WASH SOLUTION 5X (RINSE)
Temperature= RT/ Time= 1min / Volume = 2 ml / Agitation= MED / **Cycle=2** / Aspiration= YES
5- STEP 5= CONJUGATE (CONJ)
Temperature= RT/ Time= 30min / Volume = 2 ml / Agitation= MED / **Cycle=1** / Aspiration= YES
6- STEP 6= WASH SOLUTION 5x dil
Temperature= RT/ Time= 1min / Volume = 2 ml / Agitation= MED / **Cycle=2** / Aspiration= YES
7- STEP 7= (SUBUF)
Temperature= RT/ Time= 1min / Volume = 2 ml / Agitation= MED / **Cycle=2** / Aspiration= YES
8- STEP 8= SUBSTRATE (SUBS)
Temperature= RT/ Time= 30min / Volume = 2 ml / Agitation= MED / **Cycle=1**/ Aspiration= YES
9- STEP 9= WASH SOLUTION (RINSE)
Temperature= RT/ Time= 3min / Volume = 2 ml / Agitation= MED / **Cycle=2**/ Aspiration= YES

5.4 – PURGE PUMPS:

At the end of the procedure, approximately three hours, the pumps should be purged. At the prompt “READY **FOR A NEW TEST**?”, press “**NO”**;
At the prompt **“ENTER EDIT MODE?”** press **“NO**;
At the prompt **“CHECK HEAT**” press **“NO**; at the “**PURG PUMP**?” prompt, press **“YES”.**

5.5 – STARTING A PROCEDURE WITH A PREVIOUSLY SAVED PROGRAM:

1- At the prompt **“READY FOR A NEW TEST?**” press **“YES”.**
2- Using the ↑ **or** ↓ arrows, select the saved program.
3 - At the prompt**: “PUMP PADS IN PLACE”?**
4- Press **“YES”** when the stage is in position.
5- At the prompt**: “PREHEAT SYSTEM?”**
6- Press **“YES”** to begin preheating.
7- At the prompt**: “CLOSE TRAY COVER?”**
8- Press **“ENTER**”.
9- At the prompt “**PREHEAT SYSTEM 30”**

Press **“ENTER**” and the equipment will take 30 minutes to heat the Hybridization and Stringent Wash solutions. At the end of the process there will be an audible alarm.

10- At this step the wash, substrate and conjugate solutions should be prepared according to the number of samples. Review the calculations in item 5.2.1.
11- Perform the functions required by the equipment.
12- **“PUT TUBES IN BOTTLES” – PRESS ENTER**
13- **PRIME PUMP** - press “**YES**” and automatically the equipment will initialize each pump showing also the prompt “**DISPENSING**”. There are six pumps in total. At the end of each initialization, the system will prompt: **“PRIME PUMP AGAIN**?” (The prompts “**YES”** and “**NO”** will appear. When the prompt “**NO”** is pressed, The next pump will initialized automatically. At the prompt **“PRIME PUMP**?” press **“NO”. For stringent wash and hybridization solutions, perform PRIME only once with 2 ml of solution.**
14-The machine displays the prompt “**STRIP COUNT 20?”** If the test contains 20 samples press **”ENTER”.** The process of pipetting and dispensing reagents will happen automatically. After the end of the experiments follow the equipment prompts for washing hoses and switching off the equipment.

6- RESULTS AND INTERPRETATION

After the strips have been developed, they should be removed from the tray and placed on absorbent paper for 24 hours before the results are interpreted. After this period the strips should be secured together with the kit and the interpretation should be done by positioning the diagram (fig 1) next to the strip and noting the reaction line. Then, compare the reaction line with the template (fig 2) that accompanies the kit. Further information can be obtained from the package insert.

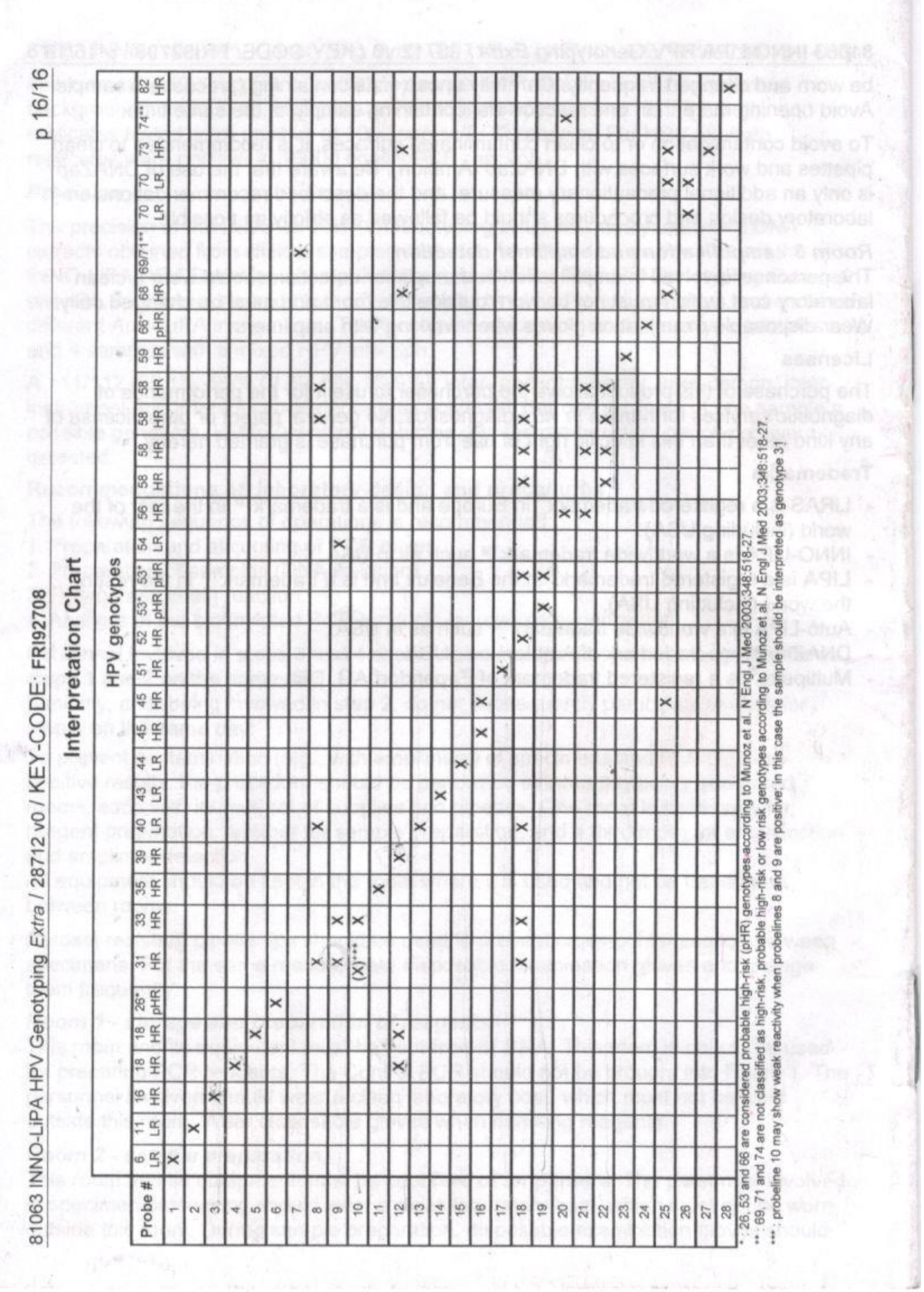

81063 INNO-LiPA HPV Genotyping *Extra* / 28712 v0 / KEY-CODE: **FRI92708** p 16/16

Interpretation Chart

HPV genotypes

Probe #	6 LR	11 LR	16 HR	18 HR	18 HR	26* pHR	31 HR	33 HR	35 HR	39 HR	40 LR	43 LR	44 LR	45 HR	45 HR	51 HR	52 HR	53* pHR	53* pHR	54 LR	56 HR	58 HR	58 HR	58 HR	58 HR	59 HR	66* pHR	68 HR	69/71**	70 LR	70 LR	73 HR	74**	82 HR
1	X																																	
2		X																																
3			X																															
4				X																														
5					X																													
6						X																												
7																													X					
8							X				X													X	X									
9							X	X												X														
10							(X)***	X																										
11									X																									
12				X	X					X																		X				X		
13											X																							
14												X																						
15													X																					
16														X	X																			
17																X																		
18							X	X			X						X		X			X	X	X	X									
19																		X	X															
20																					X												X	
21																					X		X		X									
22																						X	X	X	X									
23																										X								
24																											X							
25															X													X			X	X		
26																														X	X			
27																																X		
28																																		X

* : 26, 53 and 66 are considered probable high-risk (pHR) genotypes according to Munoz et al. N Engl J Med 2003;348:518-27.

** : 69, 71 and 74 are not classified as high-risk, probable high-risk or low risk genotypes according to Munoz et al. N Engl J Med 2003;348:518-27.

*** : probeline 10 may show weak reactivity when probelines 8 and 9 are positive; in this case the sample should be interpreted as genotype 31

Figure 1 - Diagram for interpretation of sample reactivity. The sample should be considered positive if one or more rows are positive.

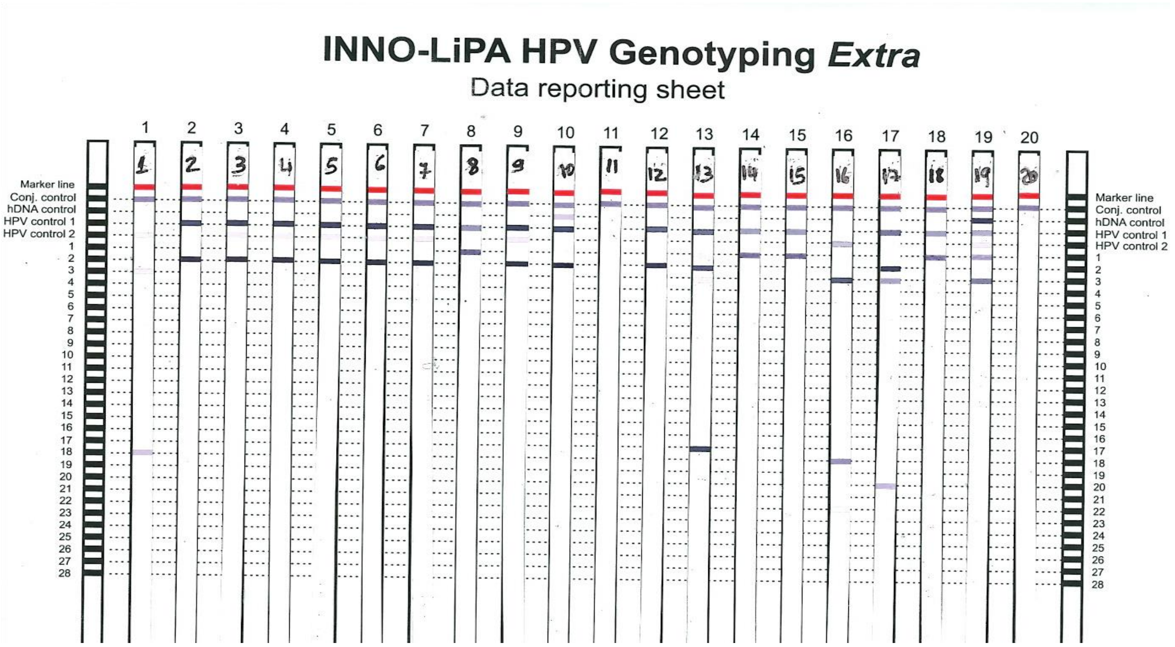

Figure 2 - Templates are located at the endpoints with reference lines including a guide line, conjugate control, for human DNA and generic controls 1 and 2 for HPV. The other lines refer to the specific location of the probes. Columns or strips from 1 to 20 are the different results from hybridizations which according to their position between 1 and 28 according to the template, should be analyzed with the help of the diagram in figure 1.
